# Non-ischemic Heart Preservation via Hypothermic Cardioplegic Perfusion Induces Immunodepletion of Donor Hearts Resulting in Diminished Graft Infiltration Following Transplantation

**DOI:** 10.3389/fimmu.2020.01621

**Published:** 2020-07-28

**Authors:** William R. Critchley, John P. Stone, Qiuming Liao, Guangqi Qin, Ivar Risnes, Andrew Trafford, Helge Scott, Trygve Sjöberg, Stig Steen, James E. Fildes

**Affiliations:** ^1^The Ex-Vivo Lab, Division of Cell Matrix and Regenerative Medicine, Faculty of Biology, Medicine and Health, Manchester Academic Health Science Centre, School of Biological Sciences, The University of Manchester, Manchester, United Kingdom; ^2^The Transplant Centre, Manchester University Hospitals NHS Foundation Trust, Manchester, United Kingdom; ^3^Department of Cardiothoracic Surgery, Lund University and Skåne University Hospital, Lund, Sweden; ^4^Department of Thoracic Surgery, Rikshospitalet, Oslo, Norway; ^5^Division of Cardiovascular Sciences, University of Manchester, Manchester, United Kingdom; ^6^Department of Pathology, Institute of Clinical Medicine, University of Oslo, Oslo, Norway

**Keywords:** heart transplantation, acute rejection, heart preservation, hypothermic cardioplegic *ex vivo* heart perfusion, passenger leukocytes

## Abstract

**Introduction:** Many donor organs contain significant leukocyte reservoirs which upon transplantation activate recipient leukocytes to initiate acute rejection. We aimed to assess whether non-ischemic heart preservation via *ex vivo* perfusion promotes immunodepletion and alters the inflammatory status of the donor organ prior to transplantation.

**Methods:** Isolated porcine hearts underwent *ex vivo* hypothermic, cardioplegic perfusion for 8 h. Leukocyte populations were quantified in left ventricle samples by flow cytometry. Cell-free DNA, cytokines, and chemokines were quantified in the perfusate. Tissue integrity was profiled by targeted proteomics and a histological assessment was performed. Heterotopic transplants comparing *ex vivo* hypothermic preservation and static cold storage were utilized to assess graft infiltration as a solid clinical endpoint.

**Results:** *Ex vivo* perfusion significantly immunodepleted myocardial tissue. The perfusate displayed a selective, pro-inflammatory cytokine/chemokine pattern dominated by IFN-γ. The tissue molecular profile was improved following perfusion by diminished expression of nine pro-apoptotic and six ischemia-associated proteins. Histologically, no evidence of tissue damage was observed and cardiac troponin I was low throughout perfusion. Cell-free DNA was detected, the source of which may be necrotic/apoptotic leukocytes. Post-transplant graft infiltration was markedly reduced in terms of both leucocyte distribution and intensity of foci.

**Conclusions:** These findings demonstrate that *ex vivo* perfusion significantly reduced donor heart immunogenicity via loss of resident leukocytes. Despite the pro-inflammatory cytokine pattern observed, a pro-survival and reduced ischemia-related profile was observed, indicating an improvement in graft viability by perfusion. Diminished graft infiltration was observed in perfused hearts compared with those preserved by static cold storage following 48 h of transplantation.

## Introduction

Heart transplantation represents the only effective treatment option for end stage heart failure, but is limited by a lack of suitable donor organs. Standard donor heart preservation utilizes static storage on ice (1), which inherently causes ischemic injury, limiting the duration for which the heart can be stored before transplantation. In an effort to address this problem and increase the donor pool, our group have developed a method of non-ischemic heart preservation using hypothermic cardioplegic *ex vivo* heart perfusion (HCP). This technology can safely extend preservation times to 24 h with stable function following transplantation (2), and has been successfully used to safely perfuse and transplant a heart by the clinical transplant team from Lund in 2017. HCP combines the protective effect of minimized metabolic demand with optimal nutritional support and oxygenation. Whilst this has clear implications for improved donor organ preservation, the potential for auxiliary benefits following transplantation have not been explored, particularly with regard to acute graft rejection.

Acute graft rejection represents a major barrier to successful transplantation requiring permanent immunosuppression, which predominantly target recipient T cells. However, little attention is paid to the donor immune compartment which can orchestrate acute rejection of the transplanted heart (3). Depletion of donor dendritic cells is sufficient to prevent rejection of transplanted lungs in mice (4) and reintroduction of donor dendritic cells restores the immune response following rat kidney transplantation (5). We have previously demonstrated that *ex vivo* perfusion is sufficient to alter immunogenicity of the donor lung and kidney via removal of passenger leukocytes, and this significantly reduces recipient T cell recruitment at 24 h post-transplantation (6, 7).

In this study, we aimed to explore the impact of HCP on the donor immune compartment and provide early pilot data to indicate how this may alter clinical outcome following transplantation.

## Materials and Methods

### Ethical Approval

The study was approved by the local Ethics Committee for Experimental Research. Animals were treated in compliance with the “Guide for the Care and Use of Laboratory Animals” published by NIH (Eight Edition, revised 2011) and the European Directive 2010/63/EU “On the protection of animals used for scientific purposes.”

## Perfusion Study

### Donor Organ Retrieval and HCP

Six healthy 6 month old Swedish pigs were used. All pigs were free of pericardial exudates and observable cardiac pathology during harvesting. Anesthesia and donor organ retrieval were performed as previously described in detail (8). HCP was performed as described previously although with continuous rather than intermittent perfusion (8). All organs were perfused at a constant 20 mmHg perfusion pressure with the aim of 100 ml/min minimum coronary flow. This fixed pressure system enables the organ to regulate its own coronary flow without forcing perfusate through at excessive pressure.

### Biopsy Processing

Left ventricle tissue was obtained by surgical dissection from the apical region before and after 8 h of HCP and split into 3 sections. Tissue weighing 30–100 mg was dissected and homogenized in 25 ml Hank's buffered salt solution (Sigma-Aldrich, Dorset, UK). Homogenates underwent serial filtration through 500, 250, and 40 μm strainers. Cells were washed and flow cytometry performed. The second section was snap frozen in liquid nitrogen and stored at −80°C. The final section was fixed in 10% buffered formalin and paraffin embedded.

### Perfusate Collection

Perfusate was collected at baseline and every 2 h throughout perfusion. The final sample was taken immediately prior to retrieval of the final myocardial sample. Samples were centrifuged at 2,000 g for 10 min and plasma stored at −80°C.

### Leukocyte Filter Processing

Following perfusion, the leukocyte filter was removed and trypsinized at 37°C for 15 min. Filter contents were assessed using flow cytometry.

### Inflammatory Profiling

Thirteen cytokines were quantified in undiluted perfusate supernatant using a porcine Luminex assay (Merck Millipore, Billerica, MA, USA) and analyzed using a Bio-Plex 200 system (Bio-Rad, Herts, UK).

### Chemokine Quantification

ELISA kits were used to quantify CCL2, CCL4, CCL5, CXCL9, CXCL10 (Insight Biotechnology, Wembley, UK) and CXCL11 (2BScientific, Oxfordshire, UK) in perfusate supernatant without dilution. Absorbance was read using a Tecan infinite 200 PRO system (Tecan Group, Männedorf).

### Flow Cytometry

Using an Attune flow cytometer (ThermoFisher, Massachusetts, US), a single cell suspension obtained by homogenization of the left ventricle was analyzed to quantify immature neutrophils (6D10+2B2–), mature neutrophils (6D10+2B2+), mature eosinophils/basophils (6D10–2B2+), helper T cells (CD4α+CD8β-), cytotoxic T cells (CD4α –CD8β+), NK cells (CD335+), B cells (CD21+), classical monocytes (CD14+CD163–), non-classical monocytes (CD14+CD163+), intermediate monocytes (CD14^dim^ CD163^bright^), and macrophages (CD203a+). SLA-DR expression was assessed as a marker of antigen presentation. Toll-like receptor 4 expression was assessed on each population. Viability was assessed using propidium iodide. All gating strategies and absolute cell counts were determined using Attune Cytometric software. Cell counts were normalized per milligram of starting tissue.

### Quantitative PCR

Primers were designed to detect mitochondrial DNA (cytochrome b) and genomic DNA (GAPDH) (Sigma Aldrich, Dorset, UK) using Primer Express® Software v3.0.1 (LifeTech, Paisley, UK) and homology assessed using BLAST (see Supplementary Methods). qPCR analysis was performed with a QuantStudio 12K Flex system using a Power SYBR green PCR master mix (LifeTech, Paisley, UK).

### Phosphokinase and Apoptosis Signaling

Tissue biopsies were obtained from each pig before and after perfusion. Phosphokinase and apoptosis antibody proteome profile arrays were used according to manufacturer's instructions (R&D systems, Abingdon, UK). A separate membrane was utilized for each sample. Chemiluminescence detection was performed using a ChemiDoc MP imaging system (Bio-Rad, Herts, UK). Pixel density analysis was performed using ImageJ (NIH, USA).

### Cardiac Tissue Viability

Troponin I was quantified in undiluted perfusate supernatant to detect cardiac injury by ELISA (Abbexa, Cambridge, UK). Absorbance was read at 450 nm using a Tecan infinite 200 PRO system (Tecan Group, Männedorf).

### Evaluation of Tissue Integrity

Histological assessment was performed using formalin-fixed tissue obtained before and after perfusion. Sections were cut at 4 μm, de-paraffinized and stained with hematoxylin and eosin. Separate sections were stained for caspase-3 expression as a marker of apoptosis (see Supplementary Methods). All samples were blinded from the consultant histopathologist.

## Heterotopic Heart Transplant Pilot Study

### Heterotopic Transplant Procedure

In order to determine whether immunodepletion by HCP altered clinical outcome we performed six heterotopic heart transplants. Six donor organs were harvested as above from 6 month old pigs. Three organs were preserved by 2 h of static cold storage and three organs preserved by 8 h of HCP. Recipient pigs (6 months old, weighing 58–64 kg) were selected based on a blood group cross match with the donor, and received anesthesia via intramuscular ketamine hydrochloride (25 mg/kg; Pfizer, Sweden) and xylasin (4 mg/kg; Bayer, Sweden). Recipient pigs were ventilated throughout the procedure. Once anesthetized, a longitudinal incision was made to the left of the linea alba. At implantation, the aorta of the donor heart was sutured end-to-side to the infrarenal aorta and the pulmonary artery was connected end-to-side to the vena cava. Reperfusion was commenced at the earliest opportunity and the hearts were defibrillated if sinus rhythm was not spontaneously established. Once the donor heart had achieved sinus rhythm, the incision was closed and the pig was awakened. Recipient pigs were maintained without immunosuppression for 48 h. Following euthanasia, biopsies were collected from the heart for histological analysis.

### Histological Evaluation of Graft Infiltration

Histological assessment of the donor heart was performed. 4 μm sections were de-paraffinized and stained with hematoxylin and eosin. Samples were prepared and assessed by a consultant histopathologist who reported intensity of leukocyte infiltration on an ordinal scale of severity (0 = no infiltration, 1 = mild infiltration, 2 = moderate infiltration, and 3 = severe infiltration). The distribution of infiltration was analyzed and presented as a percentage of the field of view affected.

### Statistical Analysis

Prism software version 7.00 (GraphPad, LaJolla, California, USA) was used to perform all statistical analyses. Data are expressed as mean ± standard deviation if normally distributed or as median [interquartile range] if non-normally distributed. Paired samples *T*-tests or related samples Wilcoxon signed rank tests were utilized to assess the changes in leukocyte content and protein expression profile between pre and post perfusion tissue samples depending upon data distribution. The related samples Friedman's two-way ANOVA by ranks was utilized to assess changes in the perfusate over time. Statistical significance was accepted when *p* ≤ 0.05.

## Results

### Baseline Myocardial Leukocyte Content

**Figure 1:**
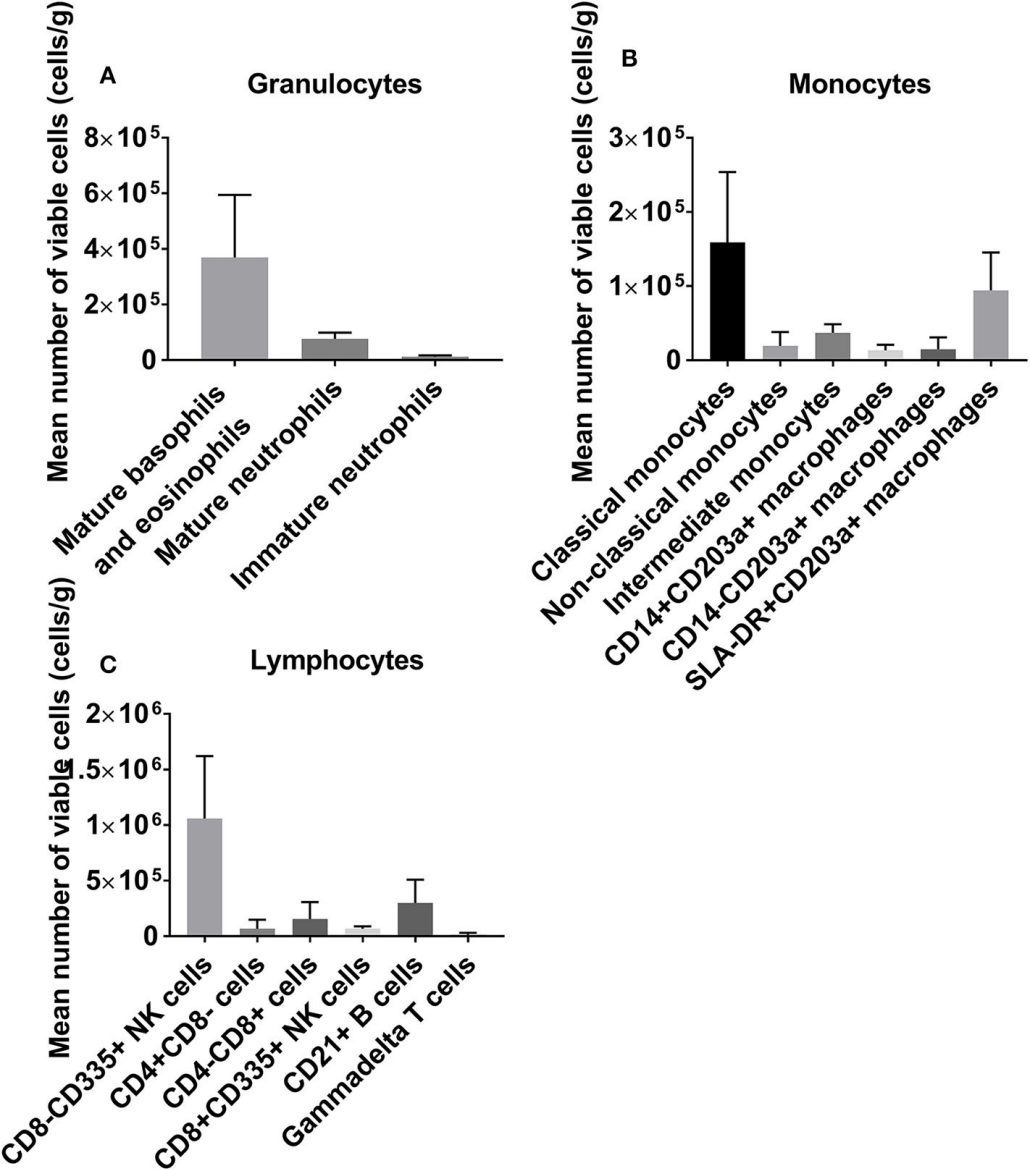
A baseline reference of the leukocyte repertoire resident within the donor heart, categorized into granulocytes **(A)**, monocytes/macrophages **(B)**, and lymphocytes **(C)**. NK cells are abundant, with large granulocyte populations and B cells also observed.

We first profiled the donor heart immune repertoire by flow cytometry to generate a baseline reference using a single cell suspension from left ventricle tissue. We demonstrate a significant cardiac-resident immune repertoire including large populations of both innate and adaptive cells. NK cells represent the largest immune phenotype detected in the tissue (Figure 1).

### Perfusion-Associated Variables

Clinically relevant parameters associated with organ retrieval and HCP were recorded. A mean cold ischemic time of 18.5 ± 7.66 min between retrieval and perfusion was recorded. Coronary flow ranged from 100 to 200 mL/min depending on organ weight.

### HCP Induces Donor Heart Immunodepletion

**Figure 2:**
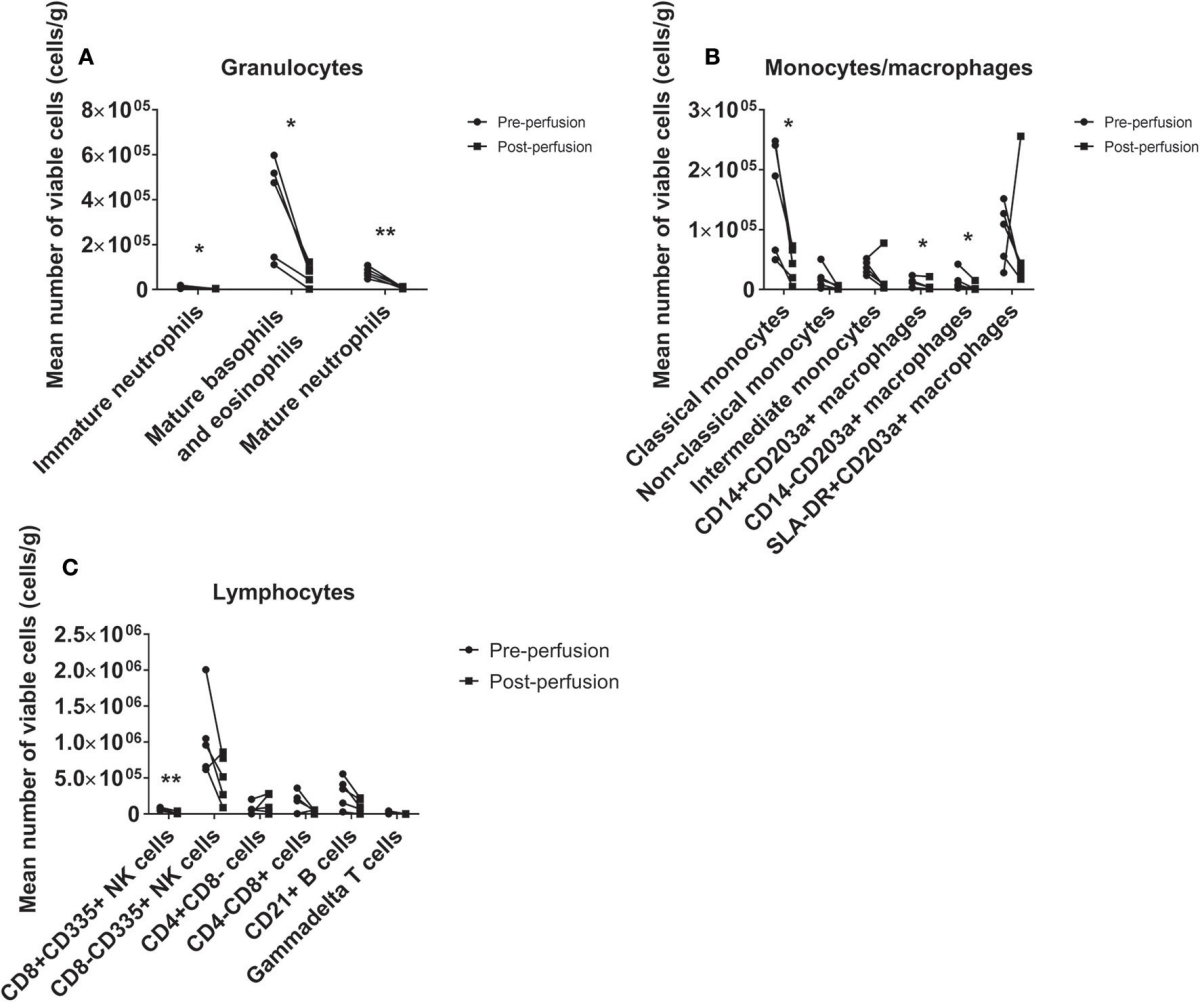
Immunodepletion of the donor heart via HCP. We observed significant leukocyte loss from the tissue across a range of phenotypes, including granulocytes **(A)**, monocytes/macrophages **(B)**, and lymphocytes **(C)**. All granulocyte populations were markedly reduced, in particular mature neutrophils and mature basophils/eosinophils (86 and 84% reductions, respectively). *p < 0.05, **p < 0.01.

There was a significant loss of viable leukocytes from the tissue following perfusion (Figure 2), including mature neutrophils (−85%, *p* = 0.003), mature basophils/eosinophils (−84%, *p* = 0.023), classical monocytes (−72%, *p* = 0.024), and B cells (−60%, *p* = 0.042). Depletion of immature neutrophils (*p* = 0.011), CD14+CD203a+ and CD14–CD203a+ macrophages (both *p* = 0.043) and CD8+ NK cells (*p* = 0.003) was also observed. Non-classical monocytes (*p* = 0.117) and γδ T cells (*p* = 0.119) were reduced for each sample pair but this did not reach significance. CD8– NK cells were markedly reduced in all but one heart although this was not statistically significant (*p* = 0.129). Intermediate monocytes (*p* = 0.225), SLA-DR+CD203a+ macrophages (*p* = 0.500), helper T cells (*p* = 0.409), and cytotoxic T cells (*p* = 0.140) were not altered by perfusion. Toll-like receptor 4 expression did not change significantly on any population except for mature neutrophils (see Figure S1).

### Immunodepletion Using Leukocyte Filtration

The content of the in-line leukocyte filter comprised predominantly NK cells, classical monocytes, mature basophils/eosinophils, and T cells. Whilst B cells represented a large population in the tissue and were significantly depleted by perfusion, they were not well-retained by the filter suggesting some other mechanism of loss. Collectively the leukocyte filter did not account for all cells lost (Figure S2).

### HCP Mediates an Inflammatory Storm Dominated by Interferon-γ

**Figure 3:**
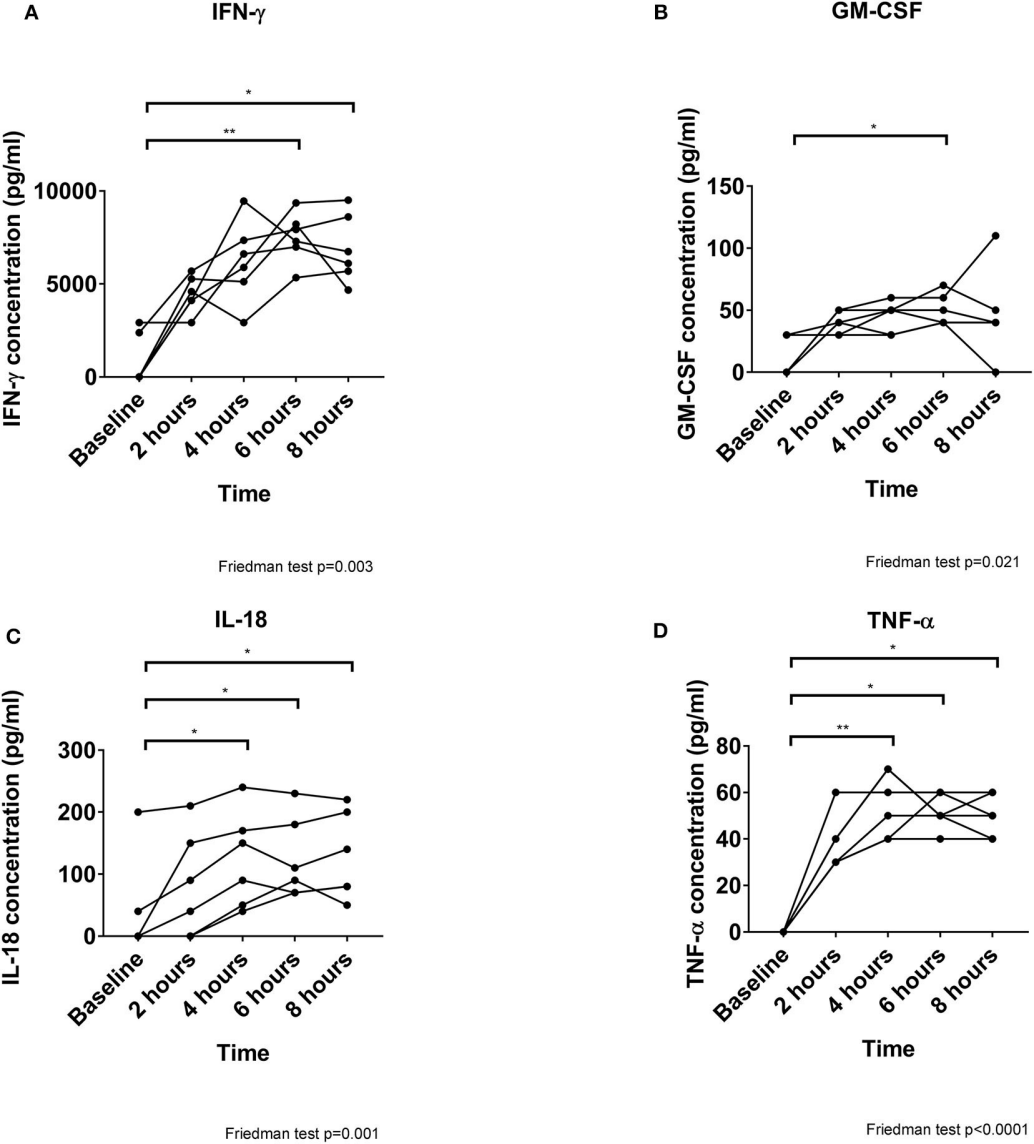
Cytokine secretion increases over time during perfusion. All 4 cytokines detected are increased significantly as perfusion progresses, although IFN-γ **(A)** is released at markedly greater concentrations than GM-CSF **(B)**, IL-18 **(C)**, and TNF-α **(D)**. *p < 0.05, **p < 0.01.

Of Twelve cytokines analyzed, only 4 were detected. Interferon-γ (IFN-γ) increased markedly and dominated the cytokine profile (peaking at 7,610 pg/ml, *p* = 0.003, Figure 3A). Significant increases in granulocyte-macrophage colony-stimulating factor (GM-CSF) (peaking at 50 pg/ml, *p* = 0.021, Figure 3B), interleukin (IL)-18 (peaking at 120p pg/ml, *p* = 0.001, Figure 3C) and tumor-necrosis factor (TNF)-α (peaking at 55 pg/ml, *p* = 0.001, Figure 3D) were also detected as perfusion progressed.

### Chemokine Release Is Induced by HCP

**Figure 4:**
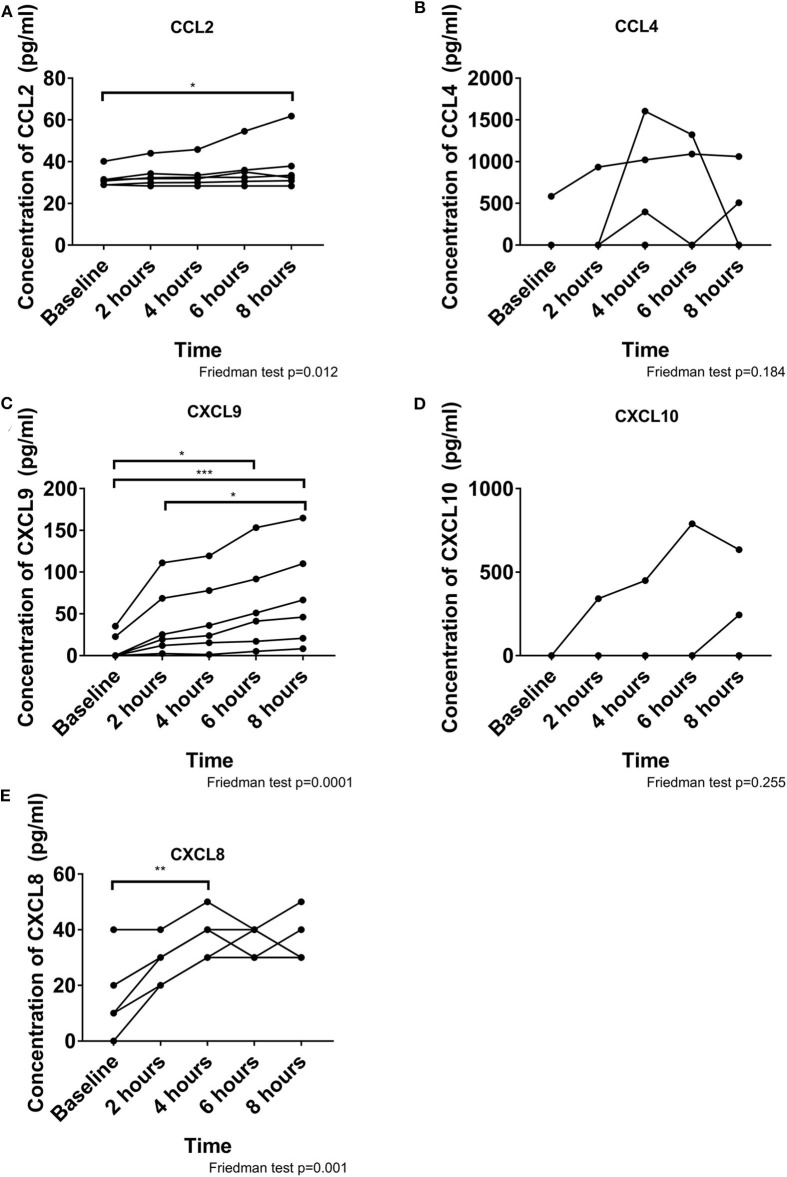
Chemokine release during perfusion is dominated by CXCL9, CXCL8, and CCL2. A small increase is observed over time for CCL2 **(A)**. CCL4 does not change over time and was inconsistent between pigs **(B)**. CXCL9 consistently increased over time **(C)**. CXCL10 was not detected in all pigs and did not change over time **(D)**. CXCL8 increased significantly by 4 hours and remained elevated throughout **(E)**. These chemokines may contribute to the migration of leukocytes out of the heart. *p < 0.05, **p < 0.01, ***p < 0.001.

To determine whether leukocyte migration occurred due to specific chemotactic signals, we quantified 7 chemokines within the perfusate (Figure 4). Due to the high IFN-γ concentration we focused on chemokines responsive to IFN-γ stimulation. CCL5 and CXCL11 were not detectable during perfusion. CXCL8 concentration increased significantly over time, peaking at 4 h but remaining elevated until 8 h (*p* = 0.001). A small increase in CCL2 (*p* = 0.021) and a large increase in CXCL9 (*p* < 0.001) were observed over time. CCL4 and CXCL10 were detected in the perfusate from 4/6 to 2/6 pigs, respectively, and thus demonstrated no significant changes over time (*p* = 0.184 and *p* = 0.255, respectively).

### Impact of Ischemia-Reperfusion Injury Following HCP

We profiled the immunodepleted tissue to assess whether HCP altered phosphorylation status of a broad range of protein kinases. Six proteins intrinsically linked to ischemia-reperfusion injury (IRI) were diminished following HCP including ^689^Y-phospho STAT2 (fold change: 0.88, *p* = 0.044), ^694^Y/^699^Y-phospho STAT5a/b (fold change: 0.82, *p* = 0.011), ^694^Y-phospho STAT5a (fold change: 0.78, *p* = 0.028), ^641^Y-phospho STAT6 (fold change: 0.87, *p* = 0.009), ^133^S-phospho CREB (fold change: 0.62, *p* = 0.045), and ^60^T-phospho WNK1 (fold change: 0.61, *p* = 0.022).

### HCP Alters Cell Death Pathways

**Figure 5:**
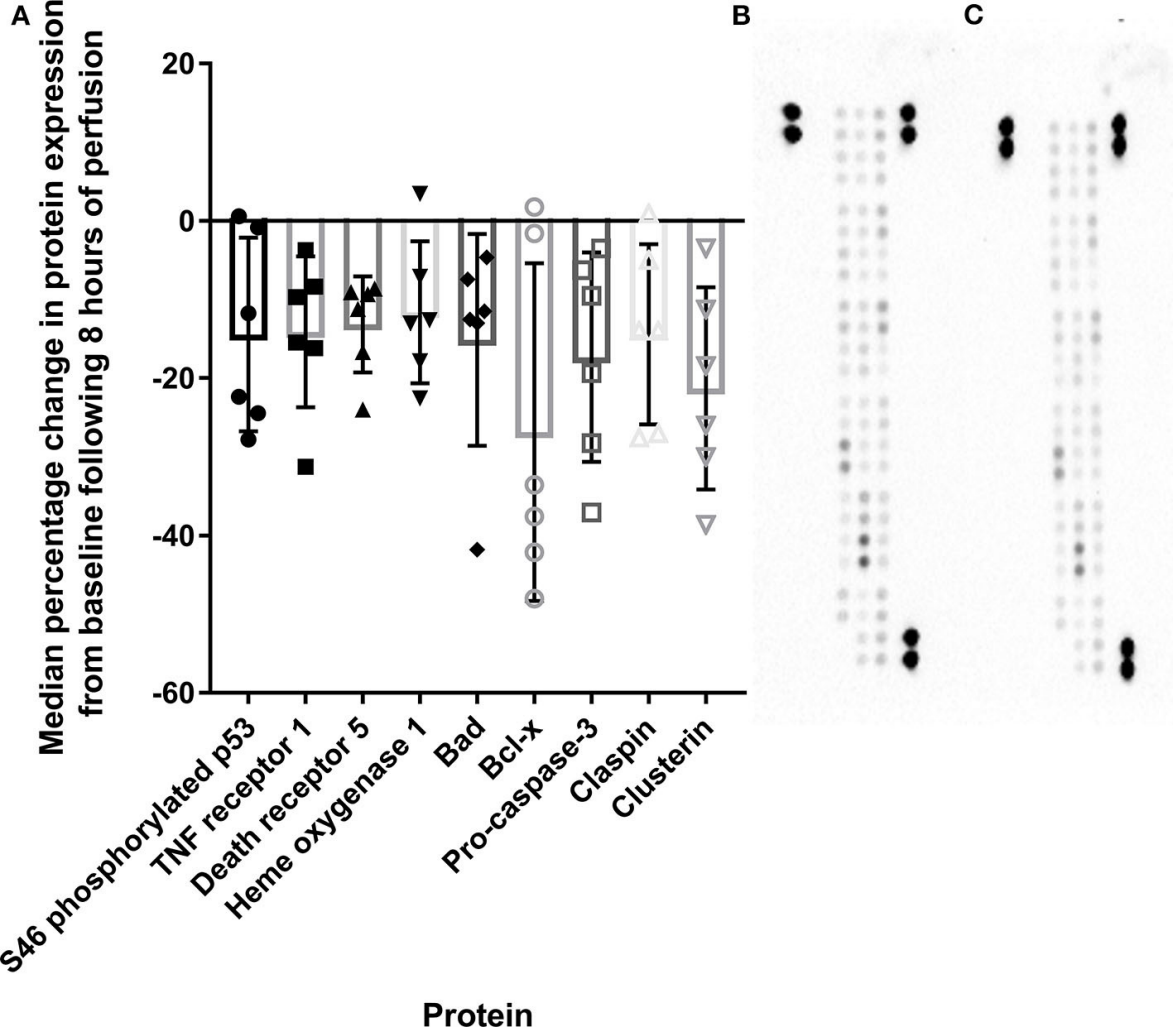
Apoptosis-related protein expression is diminished compared to baseline tissue following 8 h of HCP **(A)**. Representative array blots are provided for pre-perfusion **(B)** and post-perfusion **(C)**. TNF, Tumor necrosis factor; S46, serine 46; Bcl, B cell lymphoma.

Nine cell death proteins were diminished following HCP, including ^46^S-phospho p53 (fold change: 0.85, *p* = 0.046), TNF receptor 1 (fold change: 0.86, *p* = 0.009), death receptor 5 (fold change: 0.87, *p* = 0.001), heme oxygenase 1 (fold change: 0.88, *p* = 0.015), Bad (fold change: 0.85, *p* = 0.034), Bcl-x (fold change: 0.70, *p* = 0.041), pro-caspase-3 (fold change: 0.83, *p* = 0.019), claspin (fold change: 0.85, *p* = 0.045), and clusterin (fold change: 0.78, *p* = 0.018, Figure 5).

### Cell-Free DNA Is Released During Perfusion

**Figure 6:**
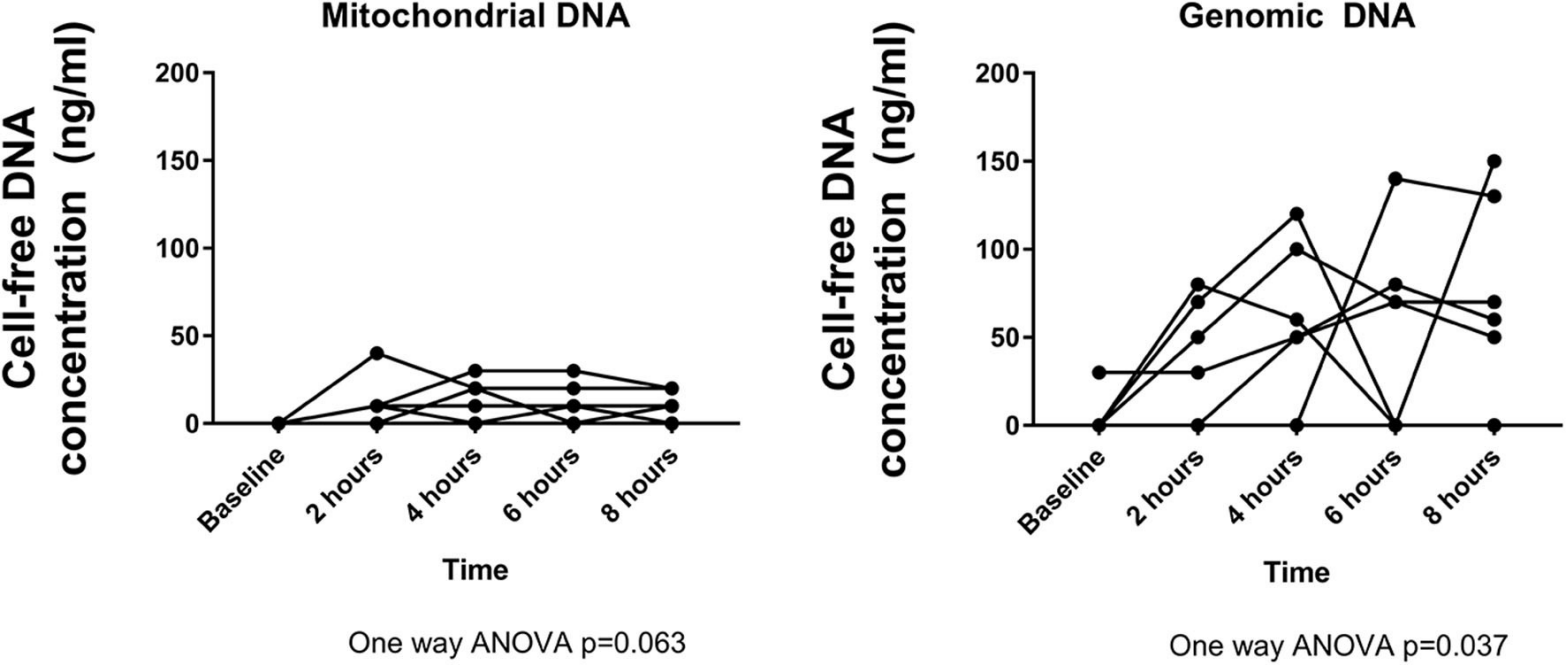
Cell-free DNA is released into the perfusate at increasing concentrations over time. Mitochondrial DNA peaks at ~4 h, whereas genomic DNA peaks at 6 h. Genomic DNA is detected at higher concentrations than mitochondrial DNA.

Steadily increasing release of both mitochondrial (*p* = 0.063) and genomic DNA (*p* = 0.037) was observed during HCP (Figure 6). Genomic DNA was consistently released at a significantly higher concentration than mitochondrial DNA (*p* = 0.009).

### Tissue Viability Is Maintained Throughout Perfusion

**Figure 7:**
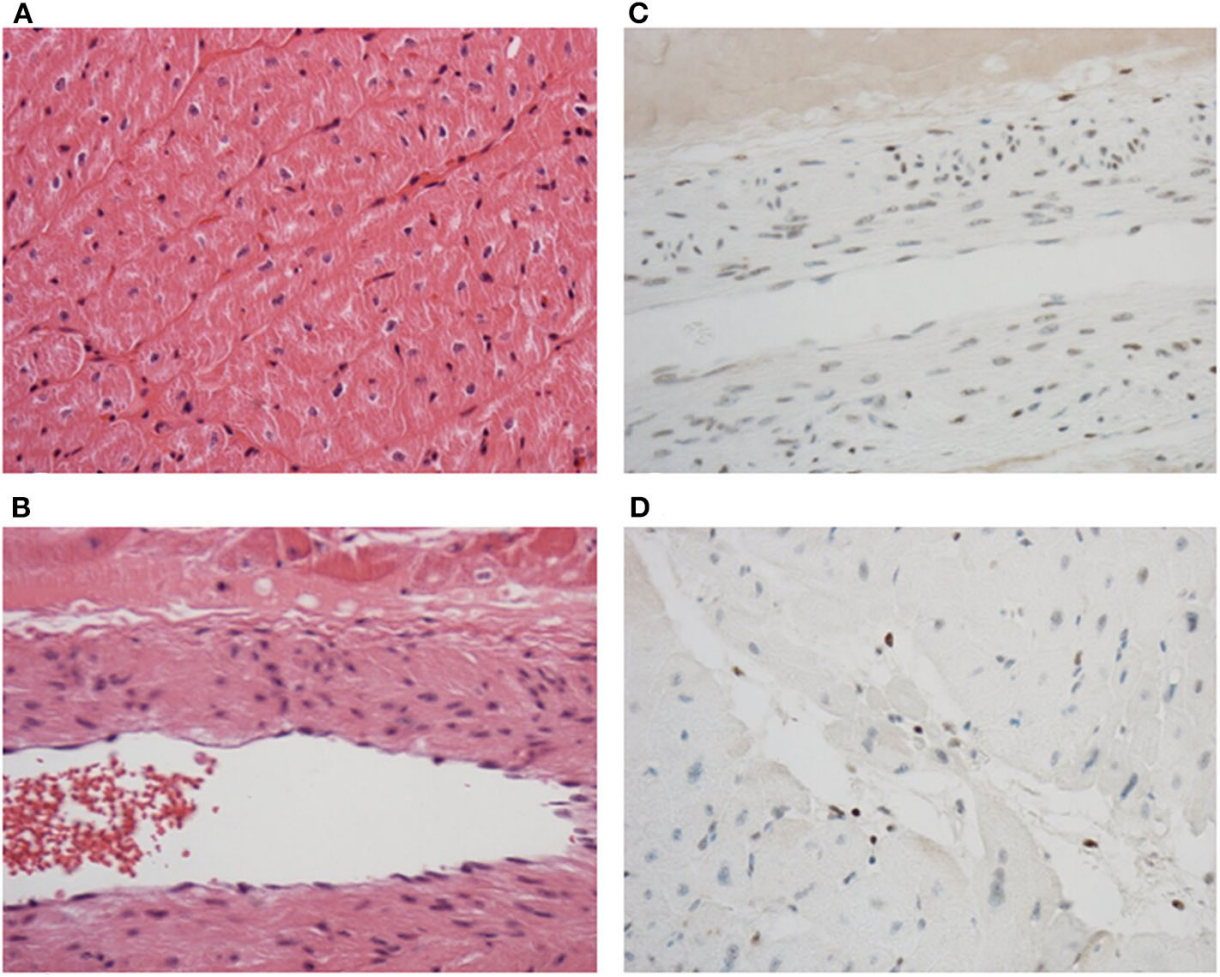
Tissue architecture and structural integrity are maintained throughout perfusion. No edema or damage to muscle **(A)** or endothelial cells **(B)** were observed after perfusion in sections stained with hematoxylin and eosin. No caspase-3 induction was observed in the muscle, endothelium, or fibroblasts **(C)**, but was detected in leukocytes **(D)** in sections stained by immunohistochemistry to detect caspase-3. Discrete brown staining in **(D)** indicates caspase-3 positive leukocytes. Original magnification was 200 × **(A)** and 400 × **(B–D)**.

As a clinically relevant end-point, a blinded histological analysis of tissue architecture was performed. HCP preserved the myocardium without ischemia or endothelial disruption (Figure 7) and caspase-3 expression remained undetectable in the muscle, endothelium, and fibroblasts, although apoptotic leukocytes were observed (Figure 7). All hearts were deemed suitable for transplant following perfusion.

### HCP Is Not Associated With Myocardial Injury

Cardiac troponin I remained stable during perfusion [median (IQR); baseline: 0.00 (71.16), 2: 0.00 (53.83), 4: 27.31 (61.56), 6: 0.00 (78.01), 8: 0.00 (86.75) pg/ml, *p* = 0.930] and undetectable at 8 h in 4/6 hearts.

### HCP Diminishes Post-transplant Graft Infiltration

**Figure 8:**
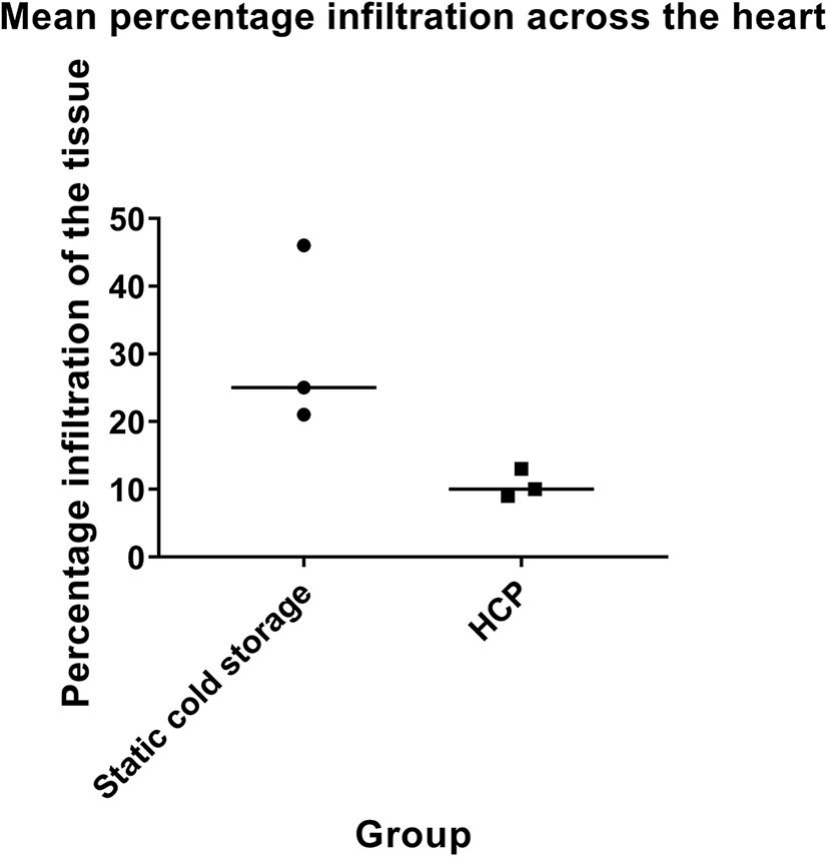
Cold cardioplegic perfusion is associated with a reduction in leukocyte infiltration at 48 h. Intensity of leukocyte infiltration is also reduced by HCP. Donor hearts preserved by cold cardioplegic perfusion were deemed to have only mild infiltration across the heart, whereas those preserved by static cold storage were considered to have moderate infiltration. Data is displayed for *n* = 3 transplants in each group.

A series of six heterotopic transplants were performed to determine if perfusion (*n* = 3 transplants) reduces recipient leukocyte recruitment into the graft post-transplantation compared with static cold storage (*n* = 3 transplants). HCP was associated with diminished graft infiltration compared to static cold storage as determined by percentage of the total cardiac tissue affected (cold stored vs. perfused: 30.7% ± 13.4 vs. 10.7% ± 2.1, *p* = 0.06, Figure 8). This was true for distribution of leukocytes within the coronary arteries (cold stored vs. perfused: 43.3% ± 23.1 vs. 14.0% ± 10.4), left ventricle (cold stored vs. perfused: 35.0% ± 39.1 vs. 6.7% ± 2.9), right ventricle (cold stored vs. perfused: 26.7% ± 22.5 vs. 11.7 ± 11.6), and septum (cold stored vs. perfused: 18.3% ± 5.8 vs. 11.7% ± 7.6). Alongside the effect on tissue distribution, the intensity of the infiltration was also diminished by HCP. Overall intensity of infiltration for perfused donor hearts was mild whereas overall intensity in the cold stored hearts was moderate.

## Discussion

Allograft rejection occurs via recipient T cell priming and infiltration of the heart. Whilst current therapies predominantly target recipient T cells, immunomodulation at an earlier stage may be advantageous. We have previously demonstrated a significant role for passenger leukocytes in the induction of T cell alloreactivity following lung transplantation (7). However, it remained unclear whether similar benefits would be observed in other organs with less well-defined resident immune repertoires.

We describe herein that the donor heart possesses a broad leukocyte compartment which could contribute to acute rejection. Donor leukocytes traffic to recipient lymph nodes upon revascularization and prime recipient allospecific T cells as part of direct allorecognition. Previous studies of cardiac donor immunodepletion have provided distinct benefits suggesting that removal of passenger leukocytes is broadly advantageous. In animal models, the specific depletion of donor CD4 T helper cells by anti-CD4 antibody or irradiation is associated with ameliorated recipient alloresponse as these cells were shown to contribute to cardiac allograft vasculopathy (9). This effect was further supported by subsequent work from the same group (10). A murine cardiac transplant study also demonstrated a role for donor passenger lymphocytes in augmenting the alloresponse to the cardiac graft, resulting in early graft failure and vasculopathy (11). In our study, we demonstrate that perfusion is sufficient to induce the depletion of a significant proportion of the donor immune repertoire prior to transplantation. Prior studies have demonstrated that donor-derived regulatory T cells are beneficial in prolonging allograft survival and thus their removal may be undesirable (12). Whilst we were unable to evaluate regulatory cells in the current study, it is likely that these cells will also have been lost from the graft. However, HCP was associated with a clinically relevant reduction in recipient leukocyte recruitment of the donor heart until at least 48 h post-transplantation in our pilot study, without any immunosuppression when compared to storage on ice. Indeed, the level of graft infiltration was diminished by ~65% relative to that in the control group, suggesting a marked beneficial effect despite this potential loss of regulatory cells. It is important to note that whilst there was consistently lower levels of infiltration in the HCP group, this only reached statistical significance at the 10% level due to the low number involved in the pilot arm of the study. This provides novel evidence that HCP drives immunodepletion and alters alloreactivity without the requirement for immunosuppression. HCP allows continuous coronary flow that promotes the clearance of passenger leukocytes from the donor organ. The extent of this flow is important in maintaining healthy myocardial tissue, and no perfused organs in this study displayed any indications of ischemia or other damage to suggest interrupted or insufficient flow. Whilst we did not evaluate the extent to which the level of coronary flow (either during perfusion or post-transplant) corresponds to intensity of subsequent graft infiltration, it is apparent that some level of coronary flow during storage is necessary for the benefits we observed. Our method therefore provides the dual-benefits of removal of passenger leukocytes alongside the extended safe storage of the donor organ as described previously.

The cause of this immunodepletion is unclear but may be in response to the inflammatory milieu in the perfusate, particularly IFN-γ. High IFN-γ concentrations were unexpected from an isolated donor heart, but further emphasize the role of the donor immune response in the immediate events following transplantation. Potentially, HCP may “therapeutically exhaust” this IFN-γ response, reducing inflammation post-transplantation. IFN-γ is not directly chemotactic but induces CCL2, CXCL8, and CXCL9 secretion (13). The release of such a milieu of IFN-γ associated proteins during HCP suggests a prominent role for this signaling network in mediating leukocyte migration from the heart. This is particularly true for CXCL9 which if neutralized, prevents IFN-γ secretion and is essential for donor specific T cell reactivity (14).

The distinct cytokine pattern observed here is interesting as many of these proteins have been previously implicated in transplant rejection. Prior analysis of cytokine levels following transplantation has demonstrated that IFN-γ is highly upregulated during rejection (15) and allograft survival is prolonged when its production is suppressed (16). Further, NK cells devoid of IFN-γ have diminished ability to induce lesions as part of antibody-mediated chronic allograft vasculopathy, illustrating the importance of minimizing transfer of this cytokine to the recipient (17). IL-18 has a well-documented role in promoting the lymphocytic production of IFN-γ, and as such IL-18 elevation in the perfusate is consistent with the massive IFN-γ levels observed (18). Neutralization of IL-18 provides protection for the cardiac allograft in murine models, as indicated by significantly prolonged survival, suggesting a key role (19). Both GM-CSF and TNF-α have been shown to be elevated during rejection (15) and GM-CSF is known to promote the differentiation of pro-inflammatory dendritic cells, which could potentially enhance alloantigen presentation (20). If the perfusate provides an approximate indication of the levels of cytokines that could be released from passenger leukocytes post-transplant then there are obvious benefits to ensuring that these are released in the circuit rather than in the recipient.

Aside from the impact of HCP on donor immunity, we also report that perfusion maintained tissue viability with no observable edema or endothelial damage. This was accompanied by minimal cardiac troponin I release. Moreover, tissue obtained post-perfusion displayed a molecular signature indicative of reduced apoptosis and IRI compared with corresponding tissue taken at retrieval following cardioplegia. HCP alone reduced STAT5 and STAT6 pathway activation which contributes to myocardial injury following ischemia and reperfusion (21, 22). Hypothermic preservation of rat donor hearts with continuous perfusion of mesenchymal stem cell conditioned medium was previously shown to protect against IRI (23). This was associated with diminished pro-inflammatory cytokine expression and increased levels of the anti-oxidant superoxide dismutase-2. It is difficult to compare these results with those presented in our study due to differences in perfusate used. However, it does provide insight into potential additional factors that could be supplemented to further bolster the beneficial effects of hypothermic perfusion. Whilst the changes in protein expression induced by HCP in our study are beneficial immediately prior to transplant, it is difficult to discern whether this altered profile reflects changes in protein expression on the myocardium or reflects loss of signals due to immunodepletion. However, caspase-3 was not evident histologically in endothelium, cardiomyocytes, smooth muscle, or fibroblasts, but was identified in remaining tissue leukocytes, supporting leukocyte death as the source of cell-free DNA during HCP.

Principally designed as a method of extending the safe storage of the donor heart, it has now become apparent that there are auxiliary benefits to organ preservation in this manner. Further studies are necessary to determine whether these proposed benefits are translated into the clinic and such a trial is currently underway. There are also additional uses that may be further explored including the possibility of organ reconditioning during perfusion, which has been postulated to occur in other systems (2, 24, 25). The device may also be utilized as a platform for delivery of therapeutic agents, which could be added for the duration of storage but flushed out from the vasculature prior to transplantation if necessary.

## Limitations

The donor animals were healthy and did not undergo brain or circulatory death as would be the case for standard donation. This may increase the immune content of the organ prior to retrieval. Due to the lack of porcine specific antibodies, we could not investigate the impact of perfusion on these phenotypes nor identify leukocytes within the tissues using immunohistochemistry. Furthermore, we could not determine the source of cell-free DNA within the perfusate. However, we suggest that leukocyte apoptosis/necrosis may be a major contributor as we observe no damage to the graft but do note significant leukocyte loss from the tissue combined with observable caspase-3 expression in leukocytes. The number of transplantations performed in the pilot arm of the study was low in keeping with NC3Rs principles, meaning a low power to detect a statistically significant difference, although the numerical difference detected between the groups was profound. We also limited survival to 48 h in the interests of the welfare of the animals.

## Conclusion

Collectively, this study reinforces the importance of the donor as a therapeutic target for immunomodulation. It also provides evidence that HCP alters the immune content and molecular signature of the donor heart, which in turn reduces recipient T cell recruitment up to 48 h following transplantation in the absence of immunosuppression. Incorporating HCP into clinical practice could potentially allow the use of more immunosuppression-sparing regimens. These exciting findings require translation with discarded human tissue prior to incorporation into clinical practice, although the technique clearly holds great promise for revolutionizing donor heart storage.

## Data Availability Statement

All datasets generated for this study are included in the article/Supplementary Material.

## Ethics Statement

The animal study was reviewed and approved by Malmö/Lunds regionala djurförsöksetiska nämnd (REB). Ethic approval M174-15 Affiliation: Department of Cardiothoracic Surgery, Lund University and Igelösa Life Science AB.

## Author Contributions

WC, JS, and HS participated in research design, writing of the paper, performance of the research, and data analysis. QL, GQ, and IR participated in performance of the research. AT participated in research design and writing of the paper. TS, SS, and JF participated in generating the original concept, research design, writing of the paper, performance of the research, and data analysis. All authors contributed to the article and approved the submitted version.

## Conflict of Interest

The authors declare that the research was conducted in the absence of any commercial or financial relationships that could be construed as a potential conflict of interest.

## Supplementary Material

Figure S1 Toll-like receptor 4 expression is significantly reduced on the surface of mature neutrophils following HCP compared to pre-perfusion values (*p* = 0.004). No changes in surface expression of toll-like receptor 4 were observed for mature basophils/eosinophils (*p* = 0.500), immature neutrophils (*p* = 0.500), CD335+CD8– NK cells (*p* = 0.893), CD335+CD8+ NK cells (*p* = 0.933), B cells (*p* = 0.074), helper T cells (*p* = 0.526), cytotoxic T cells (*p* = 0.938), γδ T cells (*p* = 0.715), classical monocytes (*p* = 0.819), non-classical monocytes (*p* = 0.152), intermediate monocytes (*p* = 0.434), CD14+CD203a+ macrophages (*p* = 0.686), CD14–CD203a+ macrophages (*p* = 0.579), or SLA-DR+CD203a+ macrophages (*p* = 0.801).

Figure S2 Immune populations are sequestered by the leukocyte filter. The leukocyte pattern detected in the filter reflects that observed in the baseline reference of the donor heart and includes granulocytes **(A)**, monocytes/macrophages **(B)**, and lymphocytes **(C)**.

## Abbreviations

HCP: Hypothermic cardioplegic *ex vivo* heart perfusion
DNA: Deoxyribonucleaic acid
IFN: Interferon
CD: Cluster of differentiation
GAPDH: Glyceraldehyde-3-phosphate dehydrogenase
STAT: Signal transducer and activator of transcription.
