# Supplementary figures and images for: Non-ischemic Heart Preservation via Hypothermic Cardioplegic Perfusion Induces Immunodepletion of Donor Hearts Resulting in Diminished Graft Infiltration Following Transplantation
Source: Front Immunol. 2020 Jul 28;11:1621. doi: 10.3389/fimmu.2020.01621 (PMC7399062; doi:10.3389/fimmu.2020.01621)

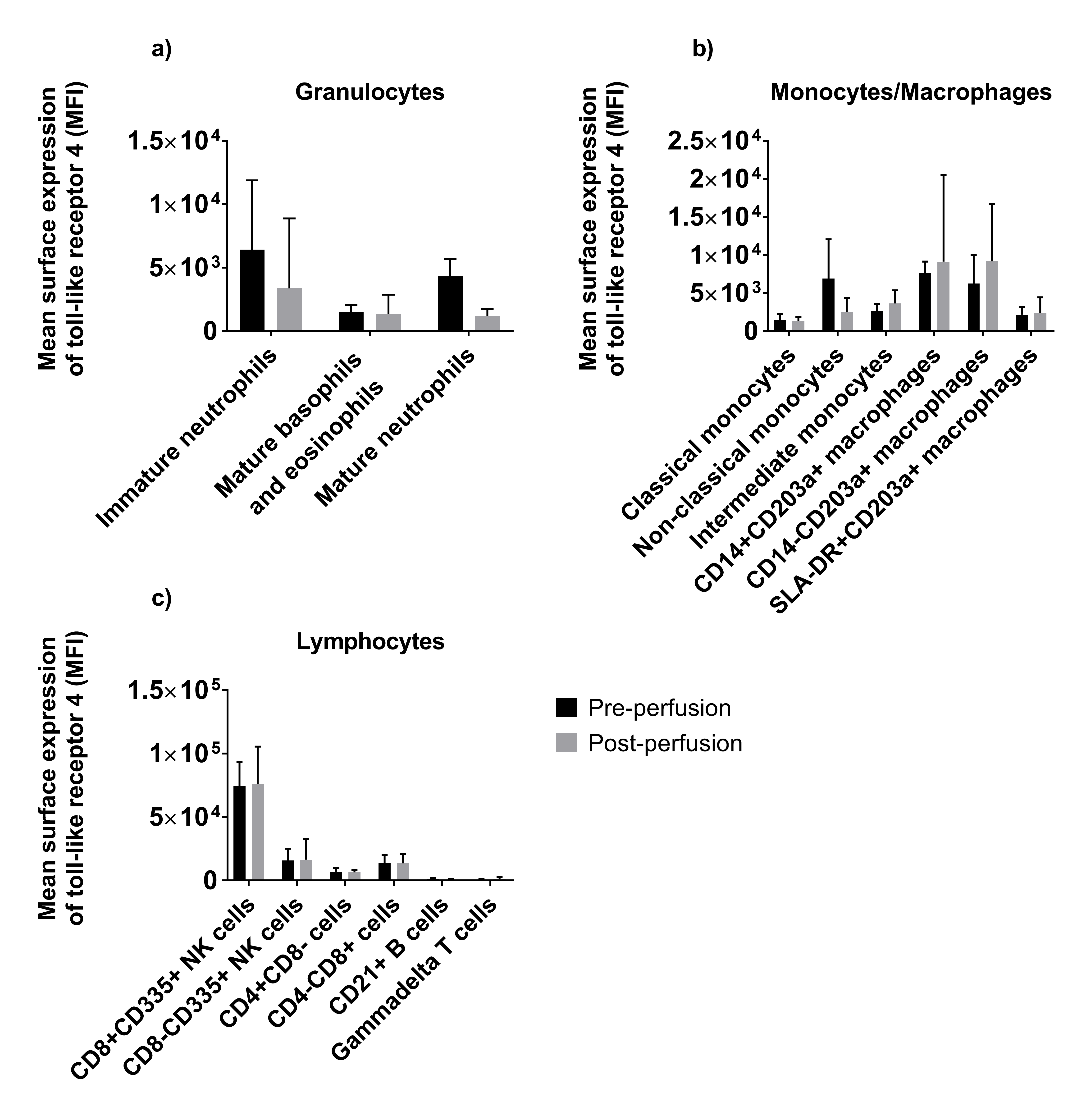

Supplement: Figure S1 — Toll-like receptor 4 expression is significantly reduced on the surface of mature neutrophils following HCP compared to pre-perfusion values (p = 0.004). No changes in surface expression of toll-like receptor 4 were observed for mature basophils/eosinophils (p = 0.500), immature neutrophils (p = 0.500), CD335+CD8– NK cells (p = 0.893), CD335+CD8+ NK cells (p = 0.933), B cells (p = 0.074), helper T cells (p = 0.526), cytotoxic T cells (p = 0.938), γδ T cells (p = 0.715), classical monocytes (p = 0.819), non-classical monocytes (p = 0.152), intermediate monocytes (p = 0.434), CD14+CD203a+ macrophages (p = 0.686), CD14–CD203a+ macrophages (p = 0.579), or SLA-DR+CD203a+ macrophages (p = 0.801). [file Image_1.TIF]

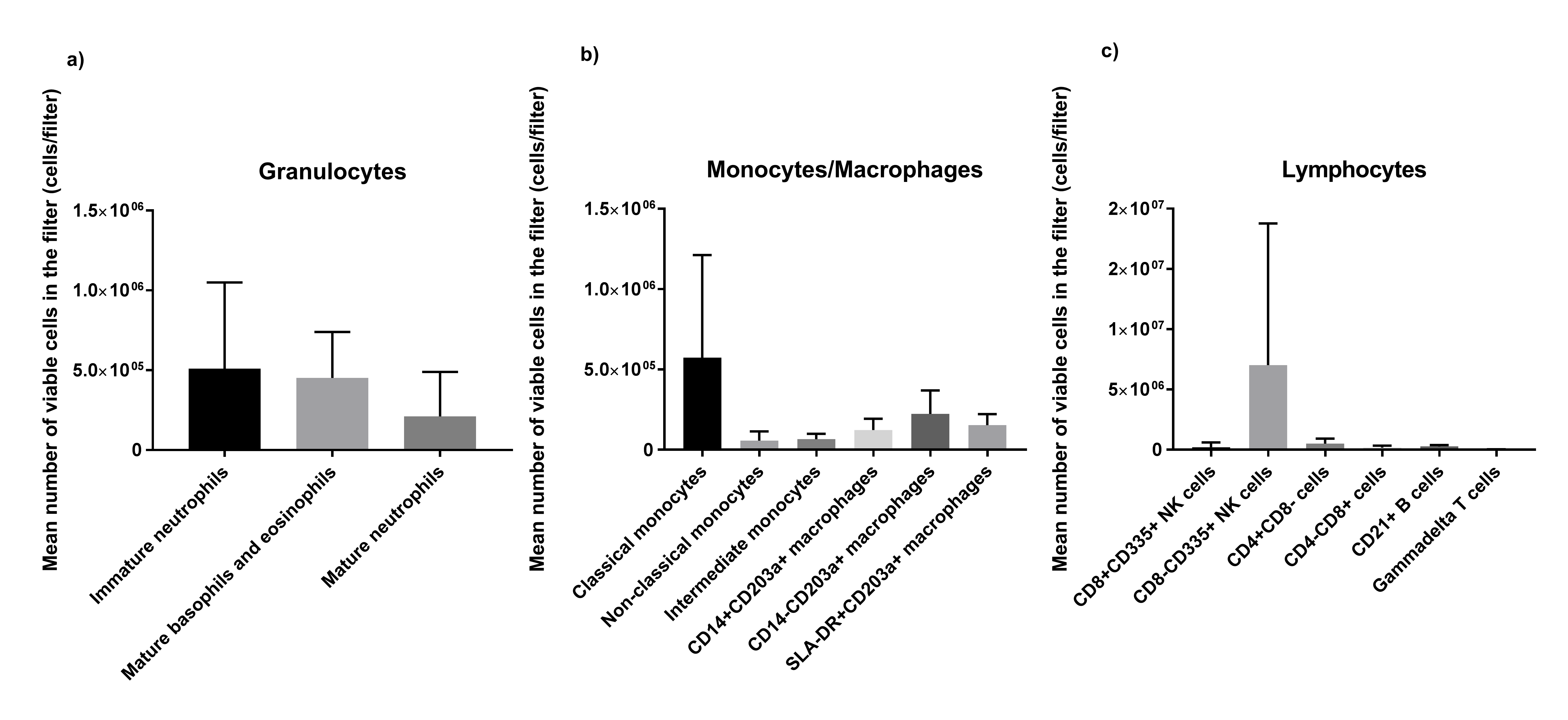

Supplement: Figure S2 — Immune populations are sequestered by the leukocyte filter. The leukocyte pattern detected in the filter reflects that observed in the baseline reference of the donor heart and includes granulocytes (A), monocytes/macrophages (B), and lymphocytes (C). [file Image_2.TIF]
