# Supplementary material for: Non-ischemic Heart Preservation via Hypothermic Cardioplegic Perfusion Induces Immunodepletion of Donor Hearts Resulting in Diminished Graft Infiltration Following Transplantation
Source: Front Immunol. 2020 Jul 28;11:1621. doi: 10.3389/fimmu.2020.01621 (PMC7399062; doi:10.3389/fimmu.2020.01621)
Supplement: Supplementary file 3 [file Data_Sheet_1.docx]

**Supplementary methods:**

qPCR:

The following primers were used:

GAPDH forward: 5’ TGCTCCTCCCCGTTCGA 3’

GAPDH reverse: 5’ GGCTTTACCTGGCAATGCA 3’

Cytochrome b forward: 5’ ACACATCAGACACAACAACA 3’

Cytochrome b reverse: 5’ GTAGCGAATAACTCATCCGTAA 3’

GAPDH and cytochrome b primers were resuspended and adjusted to 150nM and 50nM respectively using nuclease-free water (Ambion, USA).

Immunohistochemistry:

Sections were de-paraffinised and antigen retrieval (Tris-EDTA pH 9.0 buffer for 3 minutes at 750W and 20 minutes at 900W in a Whirlpool Talent Microwave Oven) was performed prior to 30 minute incubation at 37°C with the rabbit polyclonal anti-porcine caspase 3 antibody (Abcam, Cambridge, UK), diluted 1:100 in Ventana Antibody Diluent (Ventana Medical System, Tucson, USA). Binding of primary antibody to the tissue samples was detected using the Ultra View Universal DAB Detection Kit (Ventana Medical System, Tucson, USA). The slides were washed in Ventana APK detergent between incubations.
